# Coexistence of acid sphingomyelinase deficiency type A/B and Arnold-Chiari malformation: a novel case report

**DOI:** 10.3389/fped.2025.1649007

**Published:** 2025-08-25

**Authors:** Aelita Kamalova, Razilya Rakhmaeva, Gulnara Sageeva, Rezeda Safiullina, Adelina Raimova, Elena Gaichik, Dalal Nasr, Ayman A. Gobarah, Ahmed Arafat

**Affiliations:** ^1^ Pediatrics Department, Kazan State Medical University of the Ministry of Health of the Russian Federation, Kazan, Russia; ^2^ Pediatric Department of the State Autonomous Heath Institution, Children’s Republican Clinical Hospital (CPCH) of the Ministry of Health of the Russian Federation, Kazan, Russia; ^3^ Pediatrics Department, Kids Heart Medical Center, Abu Dhabi, United Arab Emirates; ^4^ Pediatrics Department, Faculty of Medicine, Suez Canal University, Ismailia, Egypt; ^5^ Pediatrics Department, Dubai Academic Health Corporation, Dubai, United Arab Emirates; ^6^ Pediatrics Department, Jiahui International Hospital, Shanghai, China

**Keywords:** Arnold-Chiari anomaly, children, acid sphingomyelinase deficiency (ASMD) type A/B, lysosomal diseases, central nervous system (CNS)

## Abstract

**Background:**

Acid sphingomyelinase deficiency (ASMD) type A/B, a rare lysosomal storage disorder caused by biallelic mutations in the SMPD1 gene, presents with variable visceral and neurological manifestations. Arnold-Chiari malformation is a structural defect of the cerebellum and brainstem with distinct pathogenesis and clinical course. To our knowledge, the coexistence of these two conditions has not been previously reported.

**Case presentation:**

We report the case of a 13-year-old patient diagnosed with ASMD type A/B in combination with Arnold-Chiari type I malformation, and secondary interstitial lung disease. The case presented a diagnostic challenge due to overlapping neurological features common to both conditions. The patient exhibited isolated cerebellar signs without MRI evidence of central nervous system involvement typically associated with ASMD. These findings, along with the radiological identification of cerebellar tonsillar herniation, supported Arnold-Chiari I malformation as the primary contributor to the patient's neurological deficits.

**Conclusion:**

This is the first documented case of concurrent ASMD type A/B and Arnold-Chiari malformation. The clinical overlap in neurological manifestations complicates differential diagnosis and highlights the need for careful neuroimaging assessment in patients with ASMD presenting with atypical or progressive neurological symptoms. This unique co-occurrence may suggest a broader phenotypic spectrum or a coincidental association requiring further investigation.

## 1 Introduction

Acid sphingomyelinase deficiency (ASMD) is a rare autosomal recessive disorder characterized by impaired lipid metabolism resulting from a deficiency of the lysosomal enzyme acid sphingomyelinase. The condition arises due to pathogenic variants in the sphingomyelin phosphodiesterase 1 (SMPD1) gene (1). The estimated birth prevalence of ASMD is approximately 0.4–0.6 per 100,000 live births (2).

Deficiency of acid sphingomyelinase results in the pathological accumulation of sphingomyelin, primarily within cells of the reticuloendothelial system, including the spleen, liver, bone marrow, and lymph nodes. In neurovisceral forms of the disease, sphingomyelin accumulation may also occur within cells of the central nervous system. The clinical phenotype of ASMD type A/B is highly variable, depending on the extent and severity of organ involvement. Multisystemic manifestations are common and may affect, in addition to the aforementioned organs, the lungs, eyes, skeletal system, and cardiovascular system. ASMD type A is characterized by severe, rapidly progressive neurodegeneration accompanied by visceral involvement. Disease onset typically occurs in early infancy, with most affected children succumbing by the age of three. ASMD disease types B and A/B are marked by the development of splenomegaly during infancy or early childhood. In many patients, thrombocytopenia arises secondary to hypersplenism. Hepatic involvement may be significant, often presenting with infiltration by foamy histiocytes, hepatocellular swelling, and varying degrees of fibrosis. Additional systemic manifestations can include interstitial lung disease, growth retardation with delayed skeletal maturation in children, and ocular findings such as macular halos and cherry-red spots (1–3).

ASMD type A/B presents with visceral features similar to or more severe than those seen in type B, along with progressive neurodegeneration of lesser severity compared to type A. A period of apparently normal development is typically observed between the ages of 2 and 7 years, after which neurological symptoms gradually emerge. These may include cognitive decline, ataxia, and extrapyramidal features; however, profound neurological impairment is generally not characteristic of the A/B phenotype (3).

The severity of the disease varies significantly, largely due to the presence of more than 180 distinct mutations in the SMPD1 gene, each exerting variable effects influenced by regional genetic diversity (4).

The Arnold-Chiari anomaly, or Chiari malformation, is a group of deformities of the posterior cranial fossa and the posterior cortex (cerebellum, pons and medulla oblongata) (5). Chiari malformations are classified based on their morphology and the severity of anatomical defects identified by neuroimaging (6). The variant of the Arnold-Chiari I malformation is often an accidental “discovery” during magnetic resonance imaging (MRI) of the brain and is characterized by a favorable, low-symptom course. In the Arnold-Chiari I malformation, a prolapse of 5 mm below the foramen magnum of one or both tonsils of cerebellum is observed. A variant of the Arnold-Chiari II malformation consists of a brainstem herniation and an elevated cerebellum in addition to a herniated amygdala of the cerebellum and vermis due to open spina bifida – myelomeningocele. The Arnold-Chiari III malformation involves the insertion of the posterior cortex (cerebellum with or without a brainstem) into a low occipital or high cervical meningoencephalocele (7). Such malformation is incompatible with the life of a child, and there are descriptions of such clinical cases in the literature (8). Arnold-Chiari IV malformation is characterized by cerebellar hypoplasia and caudal displacement of the medulla oblongata. Types II through IV typically present during childhood and are frequently associated with a broad spectrum of neurological abnormalities. More recently, an additional variant Arnold Chiari type 0 has been described. This form presents with clinical features similar to type I, including frequent headaches and other neurological symptoms; however, the degree of cerebellar tonsillar ectopia in these cases is less than 5 mm, which falls below the threshold required for classification as a classical Chiari malformation (9). Clinical signs of the Arnold-Chiari anomaly may include: coordination disorders – difficulty maintaining balance, unsteady gait, problems with fine motor skills; nystagmus; progressive muscle weakness in the arms and legs; stiffness in the back, shoulders, arms or legs; scoliosis, as well as problems with the bladder and intestines (10).

## 2 Detailed case description

We present the case of a 13-year-old male patient, born from the fourth pregnancy and the first full-term spontaneous delivery. The previous three pregnancies were electively terminated in the early stages. The patient was delivered at term with a birth weight of 2,840 g and a body length of 53 cm. He cried immediately after birth and had Apgar scores of 8 and 9 at one and five minutes, respectively. The infant was breastfed from the first day of life and was discharged from the maternity hospital on the fifth day in satisfactory condition.

Developmental milestones were achieved appropriately up to the age of two years. During this period, the parents did not report any concerns, and all laboratory evaluations and imaging studies were within normal limits. At the age of two, routine examination revealed splenomegaly and thrombocytopenia. Follow-up abdominal ultrasound examinations, performed at intervals of 3–4 months, demonstrated progressive splenic enlargement, while thrombocytopenia persisted.

At the age of three, the patient was admitted for inpatient evaluation due to splenomegaly, thrombocytopenia of unknown etiology, and recurrent episodes of acute respiratory viral infections. To exclude the possibility of a hematologic malignancy, a bone marrow aspiration was performed, which revealed the presence of foamy macrophages (Niemann-Pick cells) in the myelogram. Chest radiography demonstrated a markedly increased pulmonary vascular pattern with fine-granular texture, superimposed on a background of reticular distortion and interstitial edema predominantly in the lower medial lung zones.

Subsequent chest computed tomography (CT) revealed signs of diffuse interstitial involvement and a mild enlargement of select bronchopulmonary lymph nodes. Spirometry demonstrated a slight reduction in forced expiratory volume in one second (FEV₁) at 78.68% and vital capacity (VC) at 66.79%, consistent with a restrictive ventilatory pattern. Repeat abdominal ultrasound showed splenomegaly, with the spleen measuring 95 × 51 mm (reference range: 70.9 ± 7.1 mm). These findings raised suspicion of a lysosomal storage disorder. Gaucher disease and GM1 gangliosidosis were excluded through enzymatic activity testing. A partial molecular analysis of the NPC1 gene (associated with Niemann-Pick disease type C) was performed via direct sequencing of exons 18–22, revealing homozygous polymorphic variants c.2793C>T and c.2911+28T>C, without detection of any pathogenic mutations. Acid sphingomyelinase activity was markedly reduced (0.04 nM/mg/h; reference range: 0.56–3.24 nM/mg/h), confirming a diagnosis of ASMD. Additionally, chitotriosidase activity was significantly elevated (861 nM/mg/h; reference range: 5.50–198.0 nM/mg/h). Genetic testing to identify pathogenic variants in the SMPD1 gene was recommended but had not been conducted at the time of evaluation.

At the age of 8, the patient was hospitalized for evaluation of enuresis. According to the mother, the onset of symptoms was temporally associated with significant psychological stress related to the death of a close family member and the subsequent relocation to that relative's residence. Repeat testing of enzyme activity revealed markedly reduced acid sphingomyelinase activity (0.01 mmol/L/h; reference range: 1.50–25.0 mmol/L/h). Additionally, plasma analysis demonstrated elevated levels of lysosphingomyelin and lysosphingomyelin-509.

At the age of 9, comprehensive sequencing of the SMPD1 gene (reference sequence: NM_000543.4) identified two pathogenic variants: a known missense mutation, c.847G>A (p.A283T), previously reported in the Human Gene Mutation Database (HGMD; CM042117), and a novel frameshift mutation, c.1159delT (p.C387Vfs*9), which has not been documented in HGMD. Based on these molecular genetic findings, the diagnosis of ASMD type A/B was confirmed.

**Figure 1:**
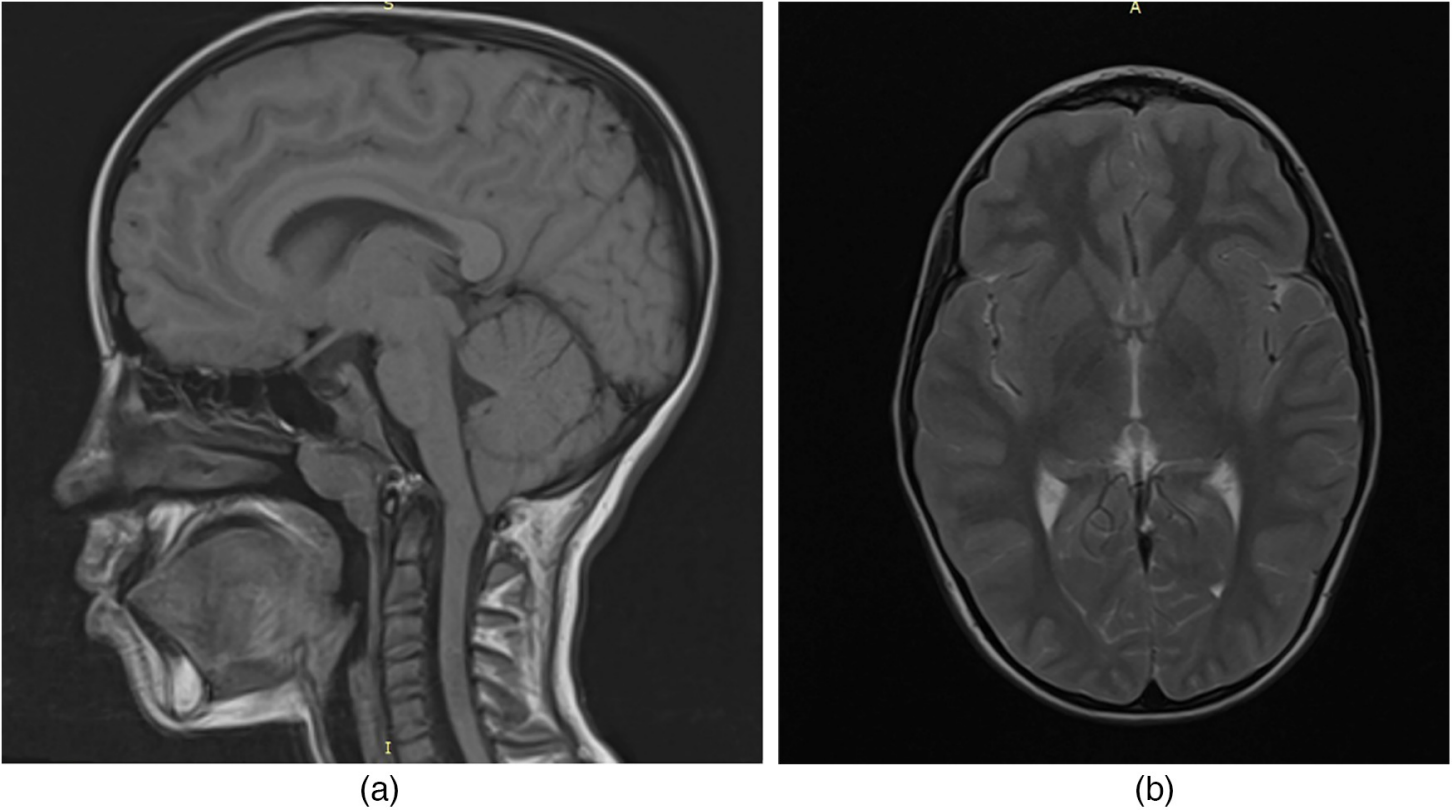
MRI of the child's brain with acid sphingomyelinase deficiency (ASMD) type A/B disease and Arnold-Chiari I malfunction: **(a)** MRI section in the sagittal plane; **(b)** MRI section in the axial plane.

To evaluate for potential central nervous system involvement, brain magnetic resonance imaging (MRI) was performed. The imaging revealed a caudal displacement of the cerebellar tonsils extending 7 mm below the foramen magnum (Figure 1), consistent with a diagnosis of Arnold-Chiari type I malformation, which was subsequently confirmed by a neurosurgical evaluation.

Assessment with the Wechsler Adult Intelligence Scale (WAIS) did not demonstrate any evidence of cognitive impairment.

At the age of 11, the patient was admitted for inpatient evaluation with complaints of recurrent epistaxis and persistent nocturnal enuresis. Coagulation studies revealed a decreased factor XII level of 38% (reference range: 50%–150%), which was not deemed clinically significant and did not require therapeutic intervention. Magnetic resonance imaging (MRI) of the lumbosacral spine excluded an organic etiology for the enuresis.

At the age of 13, the patient was re-hospitalized due to increasing fatigue, progressive splenomegaly, recurrent abdominal pain, and frequent upper respiratory tract infections. Anthropometric measurements showed a height of 152 cm and a weight of 38 kg, with corresponding *Z*-scores within the normal range (height-for-age: −0.54; BMI-for-age: −0.95), indicating average physical development. On physical examination, the liver and spleen were palpable 1 cm and 4 cm below the costal margin, respectively. The patient also exhibited hypotonia and mild signs of energy deficiency.

### 2.1 Clinical examination findings

Electrocardiography (ECG) revealed sinus arrhythmia with a heart rate ranging from 57 to 81 beats per minute and a vertical electrical axis orientation.

Ultrasound examination of the hepatosplenic system showed the liver to have a fine-grained, homogeneous parenchymal structure with normal echogenicity. Moderate perivascular hyperechogenicity was noted along the portal vein and hepatic veins. The right hepatic lobe measured 141 mm, and the left lobe measured 63 mm. The portal vein diameter was within normal limits. The spleen was enlarged, measuring 145 × 55 mm, with moderately increased echogenicity. No additional abnormalities were detected on the abdominal ultrasound.

Echocardiography demonstrated an average pulmonary artery pressure of 27 mm Hg. Cardiac chamber dimensions and hemodynamic parameters were consistent with age-appropriate norms.

Laboratory investigations revealed thrombocytopenia (platelet count: 109 × 10^9^/L) and an elevated erythrocyte sedimentation rate (ESR) of 18 mm/h. Biochemical blood analysis, arterial blood gas testing, and coagulation profiles showed no significant abnormalities.

**Figure 2:**
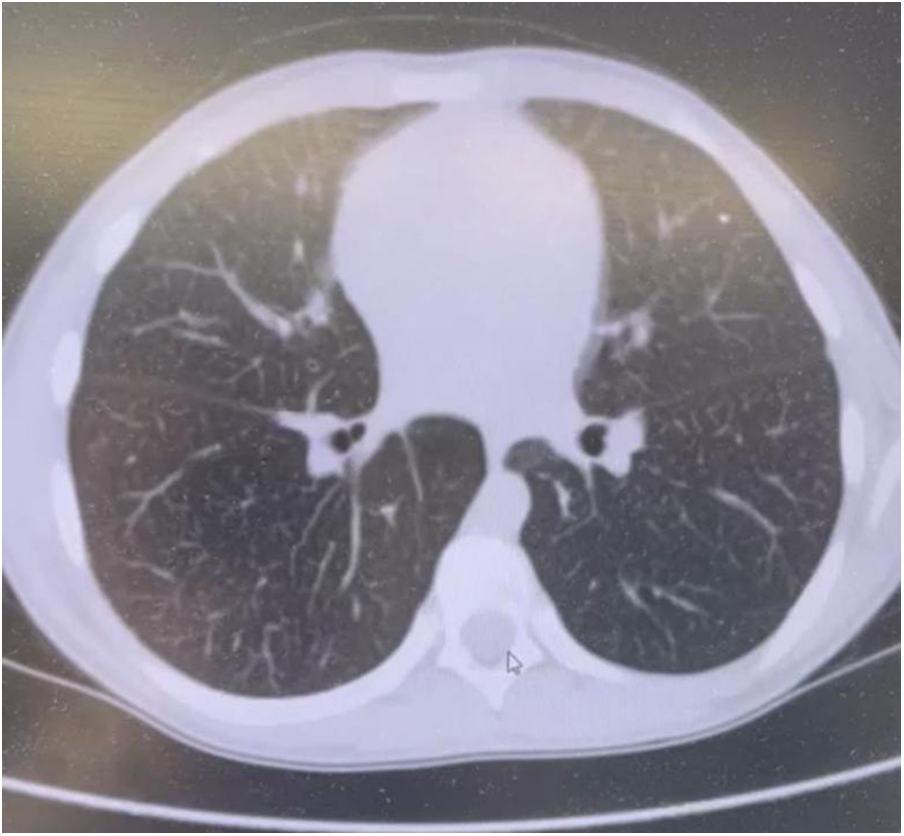
Chest CT scan of a patient with acid sphingomyelinase deficiency (ASMD) type A/B disease.

Chest computed tomography (CT) scans identified diffuse interstitial lung changes characterized by linear, reticular, and focal patterns (Figure 2).

Chest CT Scan Findings (Figure 2): Multiple linear and polygonal structures measuring up to 1–1.5 mm are observed throughout all lung fields, corresponding to irregularly thickened interlobular septa. The intralobular septa demonstrate a delicate, fine mesh-like thickening. The interstitium surrounding the vessels and bronchi exhibits mild, uneven wall thickening. Numerous small, well-defined centrilobular nodules are present in all lung zones. No evidence of acute infiltrative lung changes is noted. Pulmonary aeration is preserved without compromise. There is mild diffuse distortion of the bronchovascular architecture. Intrathoracic lymphadenopathy is characterized by multiple small paratracheal and mediastinal lymph nodes measuring up to 4 mm, alongside solitary bronchopulmonary lymph nodes measuring 11 mm on the right and 8 mm on the left. No pleural effusion is identified.

Neurological examination revealed a diffusely moderate reduction in muscle tone. The heel-to-knee test demonstrated mild dysmetria and left-sided ataxia. During the Romberg test, the patient maintained a stable stance; however, increased sway was observed in the more challenging variant of the test. Adiadochokinesia was noted predominantly on the left side, findings that, together with neuroimaging results, are consistent with Arnold-Chiari type I malformation. Cognitive assessment using the Montreal Cognitive Assessment (MoCA) test yielded a score of 26/30, indicating no evidence of cognitive impairment. Ophthalmological evaluation revealed no abnormalities. The clinical diagnosis comprised ASMD type A/B, secondary interstitial lung disease, Arnold-Chiari type I malformation, mild cerebellar dysfunction, and a mild deficiency of coagulation factor XII.

Following multidisciplinary consultation, a decision was made to initiate treatment with olipudase alfa, an enzyme replacement therapy indicated for managing the non-neurological manifestations of acid sphingomyelinase deficiency. Therapy commenced when the patient reached 14 years of age.

## 3 Discussion

The uniqueness and complexity of the presented case lie in the coexistence of two rare conditions in a child: ASMD type A/B, a hereditary lysosomal storage disorder, and Arnold Chiari malformation. The diagnosis of lysosomal storage diseases is often challenging due to their low prevalence and nonspecific clinical presentation. Although guidelines for the diagnosis and management of ASMD have been established (11, 12), a standardized clinical diagnostic algorithm or probability index for ASMD in children such as that available for Niemann Pick type C has not yet been developed (11, 12).

Moreover, genotype–phenotype correlations have been established for only a limited number of pathogenic variants. In most cases of acid sphingomyelinase deficiency (ASMD), the clinical phenotype is determined primarily by the patient's presenting symptoms and the extent of organ involvement. The diagnosis of ASMD type A/B requires a characteristic clinical picture, typically involving both neurological impairment and systemic involvement of reticuloendothelial organs such as the liver, spleen, bone marrow, and lungs. In patients exhibiting these features, measurement of acid sphingomyelinase activity is essential to confirm the diagnosis. In certain cases, reduced or absent enzyme activity is subsequently confirmed through molecular genetic testing, which identifies pathogenic variants in the SMPD1 gene, thereby establishing a definitive diagnosis of ASMD (11).

In our case, two pathogenic variants in the SMPD1 gene were identified in the patient: c.847G>A (p.Ala283Thr) and c.1159delT (p.Cys387Valfs*9).

The c.1159delT variant has been classified as pathogenic in multiple variant databases, including ClinVar (RCV000000631.5), and has been observed in patients with both type A and A/B forms of ASMD, typically associated with early-onset, severe phenotypes. The pathogenicity of this variant is consistent with the clinical presentation of the patient and supports the molecular diagnosis of ASMD. Parental testing is essential to determine whether this variant is inherited or *de novo*, which may have implications for recurrence risk and genetic counseling.

To date, only a single publication in the available literature has reported the c.1159delT mutation (13). It describes a clinical case involving a child with ASMD type A/B who exhibited neurological symptoms, including delayed motor development—specifically, the loss of postural support and the ability to roll over only after 6 months of age. The patient also demonstrated myotonic syndrome and delayed neuropsychological development, as evidenced by the inability to sit or crawl by the age of one year. Notably, this patient also carried a second mutation, c.880C>A (p.Q294), which is strongly associated with neurological involvement in ASMD. As a result, it remains unclear whether the c.1159delT mutation contributed to the observed neurological manifestations, or if these features were solely attributable to the c.880C>A variant.

Regarding the second mutation identified in our patient (c.847G>A), no definitive genotype–phenotype correlation has been established to date; however, this variant has been reported in individuals diagnosed with ASMD type B (14–16).

The importance of early and timely diagnosis of ASMD lies in the availability of disease-specific treatment with enzyme replacement therapy using olipudase alfa. This therapy was approved in 2022 by both the U.S. Food and Drug Administration (FDA) and the European Medicines Agency (EMA) for the treatment of non-neurological manifestations of ASMD. Since its approval, olipudase alfa has received marketing authorization in over 40 countries worldwide. In Russia, the drug was officially authorized for use in 2024 and was included in the “Circle of Goodness” (Russian: Круг Добра) program in 2023— a national foundation supporting children with severe, life-threatening, and chronic illnesses, including rare (orphan) diseases (17).

The most effective disease-specific therapy for acid sphingomyelinase deficiency is enzyme replacement with recombinant human ASM (olipudase alfa). Olipudase alfa significantly improves the visceral manifestations of ASMD, for example, it markedly reduces liver and spleen size and improves lung function and platelet counts (18). These improvements translate into better quality of life for treated patients. However, as a large molecule (∼75 kDa), olipudase alfa cannot cross the blood-brain barrier and therefore does not treat neurological symptoms. Thus, in chronic visceral ASMD (type B), which lacks central nervous system involvement, patients derive substantial benefit from olipudase alfa. In contrast, patients with neuronopathic ASMD (type A or A/B) who by definition have brain involvement continue to suffer neurological decline despite ERT, since olipudase alfa cannot reach the CNS (18). Recently, Geberhiwot et al. (12) published the first consensus guidelines for the clinical management of these rare disorders. Current treatment strategies include enzyme replacement therapy, solid organ transplantation (e.g., liver and lung), hematopoietic stem cell transplantation, gene therapy, and supportive symptomatic management.

In our patient, brain MRI revealed a 7 mm caudal descent of the cerebellar tonsils below the foramen magnum, consistent with Arnold Chiari malformation type I (CM-I). This form of Chiari malformation is characterized by isolated downward herniation of the cerebellar tonsils of ≥5 mm in the absence of brainstem displacement or myelomeningocele, distinguishing it from more severe forms such as type II. CM-I is often asymptomatic but can present with neurological signs including ataxia, nystagmus, vertigo, dysmetria, or headaches due to impaired cerebrospinal fluid (CSF) flow and compression of posterior fossa structures (19).

In our case, the presence of cerebellar signs, mild dysmetria, ataxia, and adiadochokinesia were observed without the characteristic CNS imaging findings typical of ASMD. These signs were therefore attributed to CM-I.

To our knowledge, the coexistence of ASMD type A/B and Arnold–Chiari malformation has not been reported previously in the literature. The two conditions likely represent a coincidental co-occurrence, given their distinct etiologies, CM-I being a congenital structural anomaly and ASMD a lysosomal storage disorder. However, it is plausible that hindbrain compression and altered CSF dynamics due to CM-I could compound or mimic ASMD-related neurological signs, particularly in patients with mild neurovisceral phenotypes. The diagnostic overlap warrants careful neuroimaging and multidisciplinary interpretation when cerebellar symptoms arise in patients with ASMD.

The coexistence of ASMD with Arnold–Chiari malformation raises the question of whether structural CNS anomalies may represent a broader, though underrecognized, feature among lysosomal storage disorders. Similar posterior fossa or craniocervical junction anomalies have been reported in other lysosomal storage disorders (LSD), such as mucopolysaccharidoses (MPS), GM1 gangliosidosis, and Niemann Pick type C (NPC). In MPS, bony dysplasia often leads to atlantoaxial instability and foramen magnum stenosis, potentially resulting in Chiari I-like presentations or cervical cord compression. Structural anomalies such as cerebellar atrophy, hindbrain herniation, and hydrocephalus have also been documented in GM1 gangliosidosis and NPC, sometimes mimicking features of Chiari malformation (20, 21).

These parallels suggest that storage-related tissue remodeling or secondary skeletal dysplasia may contribute to altered CSF dynamics and cerebellar descent in some lysosomal disorders. However, no such associations have been described to date for ASMD, which has not been traditionally linked to congenital or acquired posterior fossa abnormalities. Therefore, the co-occurrence of ASMD type A/B and Arnold–Chiari type I malformation in our patient may be coincidental but highlights the importance of neuroimaging in patients with ASMD presenting with atypical or focal cerebellar symptoms. Further case aggregation and neuroimaging studies are needed to assess whether this represents a rare extension of the ASMD phenotype.

Additionally, a recently published case report by Demircioğlu and Demircioğlu, who described a pediatric patient with ASMD type B presenting with isolated splenomegaly, a markedly different phenotype from the neurovisceral involvement observed in our case (22). This contrast emphasizes the spectrum of clinical variability in ASMD and supports the importance of genotype–phenotype correlation and individualized diagnostic pathways. We emphasize that further research is needed to understand any interaction.

## 4 Conclusion

Both Acid sphingomyelinase deficiency (ASMD) type A/B and Arnold-Chiari malformation can present with overlapping neurological manifestations, necessitating a thorough differential diagnosis. In this case, the presence of cerebellar syndrome in the absence of characteristic neuroimaging findings typically associated with ASMD suggested that Arnold-Chiari type I malformation was the primary cause of the neurological deficits. Nevertheless, given the underlying pathophysiology of ASMD, the patient requires ongoing multidisciplinary management involving both neurology and neurosurgery specialists. Regular neuroimaging follow-up is essential to monitor for disease-specific changes related to ASMD and to detect any progression of the Arnold-Chiari malformation at an early stage.

## Data Availability

The datasets presented in this article are not readily available because of ethical and privacy restrictions. Requests to access the datasets should be directed to the corresponding author.

## References

[1] Schuchman EH Desnick RJ. Types A and B Niemann-Pick disease. Mol Genet Metab. (2017) 120(1–2):27–33. 10.1016/j.ymgme.2016.12.008 28164782 PMC5347465

[2] Kingma SD Bodamer OA Wijburg FA. Epidemiology and diagnosis of lysosomal storage disorders; challenges of screening. Best Pract Res Clin Endocrinol Metab. (2015) 29:145–57. 10.1016/j.beem.2014.08.004 25987169

[3] McGovern MM Avetisyan R Sanson BJ. Disease manifestations and burden of illness in patients with acid sphingomyelinase deficiency (ASMD). Orphanet J Rare Dis. (2017) 12:41. 10.1186/s13023-017-0572-x 28228103 PMC5322625

[4] Vignesh V Kalaivani S Lokeshvaran D Aswin J Abinish DG. A rare case of type A Niemann-Pick disease and its treatment approaches. Indian J Pharmacy Practice. (2025) 18(2):239–42. 10.5530/ijopp.20250162

[5] de Arruda JA Figueiredo E Monteiro JL Barbosa LM Rodrigues C Vasconcelos B. Orofacial clinical features in Arnold Chiari type I malformation: a case series. J Clin Exp Dent. (2018) 10(4):e378–82. 10.4317/jced.54419 29750100 PMC5937955

[6] Arora R. Imaging spectrum of cerebellar pathologies: a pictorial essay. Pol J Radiol. (2015) 80:142–50. 10.12659/PJR.892878 25806100 PMC4364256

[7] Association of Neurosurgeons of Russia. Clinical Recommendations for the Diagnosis and Treatment of Chiari Malformation in Children. St. Petersburg: Association of Neurosurgeons of Russia (2015). p. 11.

[8] Ivashchuk G Loukas M Blount JP. Chiari III malformation: a comprehensive review of this enigmatic anomaly. Childs Nerv Syst. (2015) 31:2035–40. 10.1007/s00381-015-2853-9 26255148

[9] Association of Medical Geneticists, Union of Pediatricians of Russia. Clinical Recommendations: Niemann-Pick Disease Type C. Moscow: The Association of Medical Geneticists and the Union of Pediatricians of Russia (2019). p. 59.

[10] Belykh NA Khrulyova AM Piznyur IV Raeva GF. Clinical case of Arnold-Chiari syndrome in combination with dextrocardia in a child. Eur Health (2024) 5:115–21. 10.54890/1694-8882-2024-5-115

[11] McGovern MM Dionisi-Vici C Giugliani R Hwu P Lidove O Lukacs Z Consensus recommendation for a diagnostic guideline for acid sphingomyelinase deficiency. Genet Med. (2017) 19(9):967–74. 10.1038/gim.2017.7 28406489 PMC5589980

[12] Geberhiwot T Wasserstein M Wanninayake S Bolton SC Dardis A Lehman A Consensus clinical management guidelines for acid sphingomyelinase deficiency (Niemann-Pick disease types A, B and A/B). Orphanet J Rare Dis. (2023) 18(1):85. 10.1186/s13023-023-02686-6 37069638 PMC10108815

[13] Ivanova EA Vasileva TS Bushueva EV Smirnova EI Petrov AG. The course of coronavirus infection COVID-19 in a child with Niemann-Pick disease type A and B. Mod Probl Sci Educ. (2021) (3):198. 10.17513/spno.30864

[14] Hu J Maegawa GHB Zhan X Gao X Wang Y Xu F Clinical, biochemical, and genotype-phenotype correlations of 118 patients with Niemann-Pick disease types A/B. Hum Mutat. (2021) 42(5):614–25. 10.1002/humu.24192 33675270

[15] Landrum MJ Lee JM Riley GR Jang W Rubinstein WS Church DM ClinVar: public archive of relationships among sequence variation and human phenotype. Nucleic Acids Res. (2014) 42(D1):D980–5. 10.1093/nar/gkt1113 24234437 PMC3965032

[16] Zampieri S Filocamo M Pianta A Lualdi S Gort L Coll MJ Dardis A SMPD1 mutation update: database and comprehensive analysis of published and novel variants. Hum Mutat. (2016) 37(2):139–47. 10.1002/humu.22923 26499107

[17] Diaz GA Jones SA Scarpa M Mengel KE Giugliani R Guffon N One-year results of a clinical trial of olipudase alfa enzyme replacement therapy in pediatric patients with acid sphingomyelinase deficiency. Genet Med. (2021) 23(8):1543–50. 10.1038/s41436-021-01156-3 Erratum in: *Genet Med*. (2022) 24(10):2209. doi: 10.1016/j.gim.2022.08.011 33875845 PMC8354848

[18] Syed YY. Olipudase alfa in non-CNS manifestations of acid sphingomyelinase deficiency: a profile of its use. Clin Drug Investig. (2023) 43(5):369–77. Erratum in: Clin Drug Investig. 2023 43(8):667. doi: 10.1007/s40261-023-01293-4. 10.1007/s40261-023-01270-x 37133675 PMC10361862

[19] Meadows J Kraut M Guarnieri M Haroun RI Carson BS. Asymptomatic chiari type I malformations identified on magnetic resonance imaging. J Neurosurg. (2000) 92(5):920–6. 10.3171/jns.2000.92.6.0920 10839250

[20] Yilmaz S Haliloglu G Topcu M. Neurological manifestations of mucopolysaccharidoses and gangliosidoses. J Pediatr Neurosci. (2007) 2(1):13–20.

[21] Golden E van Gool R Cay M Goodlett B Cao A Al-Hertani W The experience of living with Niemann-Pick type C: a patient and caregiver perspective. Orphanet J Rare Dis. (2023) 18(1):120. 10.1186/s13023-023-02741-2 37210540 PMC10200045

[22] Demircioğlu H Demircioğlu S. A rare cause of splenomegaly: acid sphingomyelinase deficiency type B. Hitit Med J. (2025) 7(2):295–300. 10.52827/hititmedj.1624724

